# In-hospital Mortality is Lower in Brain-Injured Patients After Admission to a Neuroscience Intensive Care Unit: A Multi-Center Cohort Study

**DOI:** 10.1177/08850666251325778

**Published:** 2025-04-13

**Authors:** Angel J. Cadena-Tejada, Shaista Alam, Varoon Thavapalan, Sara Habib, Fred Rincon

**Affiliations:** 1 Department of Neurosurgery, 12340 University of Texas Health Science at Houston, Houston, TX 77007, USA; 2 Department of Neurology, 23217 Thomas Jefferson University Hospital, Philadelphia, PA, USA; 3 Department of Neurology, 5507 Aurora Health Care, Milwaukee, WI, USA; 4 Department of Neurology, 1859 Beth Israel Deaconess Medical Center, Boston, MA, USA; 5 Departments of Neurology and Neurosurgery, 2202 Cooper University Hospital, Camden, NJ, USA

**Keywords:** APACHE IV, neuro-critical care, intensive care unit, length of stay, brain injury, ventilator-free days

## Abstract

**Objective:**

To study the impact of dedicated Neuroscience Intensive Care Units (NSU) on clinical outcomes in patients with acute brain injury.

**Design:**

Retrospective, multicenter cohort study.

**Setting:**

172 intensive care units within the United States.

**Patients:**

Prospectively compiled and maintained a registry of a total of 32,047 brain-injured patients (stroke = AIS, aneurysmal-bleed = SAH, intra-cerebral-hemorrhage = ICH, and traumatic brain injury = TBI) from 2008–2013.

**Measurements:**

Exposure of interest was the type of intensive care unit (ICU), divided into NSU and non-NSU (medical = MICU, non-neurosurgical = SICU, trauma = TICU, cardiac = CCU, or mixed). Outcomes of interest were the actual and predicted in-hospital mortality, ICU mortality, ICU length of stay, and ventilator-free days. We calculated the actual and predicted in-hospital mortality using the Cerner Corporation Acute Physiology and Chronic Health Evaluation IV (APACHE Clinical Information System, CIS). We then compared the actual in-hospital mortality against the mortality prediction of the APACHE-IV model based on ICU designation (NSU v. non-NSU). The multivariable model was adjusted for within-hospital effects and known predictors of poor outcomes after brain injury.

**Main Results:**

National APACHE-IV predicted that in-hospital mortality was higher for NSU admissions than non-NSU admissions (21% v. 19%, p < .0001). However, the actual ICU mortality (10% vs 11%, p < 0.01) and in-hospital mortality (15% vs 16%, p = 0.06) were lower in patients admitted to a NSU as compared to non-NSU. We observed lower ventilator-free days (22 vs 24, p < 0.001) in NSU v. non-NSU. In the multivariable regression analysis adjusted for within-hospital effects, known variables of poor outcome, and the severity of illness APACHE-III score, the in-hospital mortality was lower for NSU admissions (OR, 0.8; 95%CI, 0.7-0.9, p = 0.02) as compared to non-NSU.

**Conclusion:**

Admission of critically ill brain-injured patients to dedicated NSUs is associated with lower actual in-hospital mortality. Future iterations of APACHE-IV modeling may need to incorporate NSU designations for calculations of expected mortality among brain-injured patients.

## Introduction

Over the past few decades, there has been an increasing interest in developing specialized intensive care units (ICUs) that provide focused care for cardiology and cardiothoracic surgery patients, neonates, trauma, cancer, and neurologic patients. Healthcare professionals certified with additional training in specialized fields provide care in respective units. Although there has been an argument about whether specialized ICUs are required since hiring specialized staff and allocating space in the hospital is costly, a few specialized ICUs have been shown to decrease hospital length of stay (LOS), reduce mortality, and improve discharge outcomes.^1,2^

In 2007, the United Council of Neurological Subspecialties (UCNS) introduced board certification in Neurocritical Care, offering two years of training to attain the title of board-certified neuro-intensivist (NI). Since then, many hospitals have built neurological/neuroscience ICUs (NSU), where neurointensivists (Nis), specialized NSU-trained nurse practitioners and physician assistants manage critically ill neurological patients. However, there are no formal recommendations or advice regarding the building and use of NSUs. Although the initial 2005 Brain Attack Coalition (BAC) consensus statement recommended that patients with acute ischemic injury should be managed by comprehensive stroke centers (CSC) with an ICU available, presence of a the dedicated NCCU and/or a formally trained NI was ‘‘desirable but not required’’.^3^ In 2011, given the growing field of neurocritical care, the BAC updated these recommendations to include that CSCs should have at least one fellowship-trained neuro intensivist on their medical staff.^4^ The American Heart Association and American Stroke Association (AHA/ASA) advise transferring patients with Intracerebral hemorrhage and aneurysmal subarachnoid hemorrhage to dedicated neurocritical care units due to the growing evidence of lower mortality rates.^5,6^

Multiple studies have demonstrated that patients that critically ill stroke patients managed by Nis and in dedicated NSUs have better outcomes in terms of discharge status and decreased hospital length of stay (LOS).^2,7–9^ A retrospective study also demonstrated a lower in-hospital mortality rate among ICH patients managed in NSUs than those managed in non-NSUs; however, longer hospital LOS was noted.^1^

To this end, we sought to explore the impact of NSU on critically ill neurological patients. We hypothesize that NSU and the presence of certified Nis and staff can have a positive outcome among critically ill neurological patients, decrease mortality and LOS in the hospital, and improve discharge disposition.

## Materials and Methods

### Study Design and Patient Population

A retrospective multi-center cohort study of 172 ICUs within the United States utilizing a prospectively compiled and maintained registry (Cerner Corporation –Acute Physiology and Chronic Health Evaluation IV [APACHE Clinical Information System, CIS], Cerner, Kansas City MO).^10^ The APACHE-IV CIS collects detailed clinical, physiological, and outcome data on adult ICU patients at participating hospitals for benchmarking (ICU and hospital mortality) and quality improvement. APACHE data are collected at each site by trained local coordinators. Specific data elements include the primary diagnosis at admission, the patient's age, location of the patient before admission to the ICU, length of hospital stay before admission, and clinical and physiological variables during the first 24 h after admission to the ICU. The APACHE-CIS has been used for numerous observational studies involving critically ill patients.^11–13^

All adult patients >17 years admitted to an ICU with a diagnosis of brain injury between 2008 and 2013 inclusive were considered eligible for the study. For this study, brain injury was defined as acute ischemic stroke (AIS), primary intracerebral hemorrhage (ICH), subarachnoid hemorrhage (SAH), and traumatic brain injury (TBI). Patients admitted after cardiopulmonary resuscitation for therapeutic hypothermia and patients with documented CNS infections (meningitis or encephalitis) were excluded from the study due to potential confounding effects. Patients with missing data for ventilator status and ventilator days, time of discharge, and condition on discharge (expired vs not) were also excluded from the study. Data fields included hospital and ICU organizational characteristics, demographics, comorbid conditions, physiological data, procedures, in-hospital complications, hospital and ICU length of stay (LOS), and discharge status. Data were collected by dedicated onsite personnel trained and certified by PI (Project Impact).

Personnel are also required to pass a written certification examination to ensure uniformity in both database definitions and entry. The ICUs in PI repository represent a broad scope of practice environments, including medical, surgical, neurological, and multidisciplinary ICUs. The institutions are heterogeneous regarding hospital size, type (community vs academic; private vs public), and location (urban, suburban, or rural). Participating hospitals are not restricted to any geographic region. Besides contributing data, Cerner Corporation made no financial or material contribution to this study. Based on the de-identified nature of the database, the Institutional Review Board of Thomas Jefferson University Hospital (Philadelphia, PA) exempted this analysis from full review.

### Definition of Main Exposure and Outcome Variables

The ICUs were stratified into two main exposure groups, NSU (Neurology, Neurosurgery, or mixed [neurology + neurosurgery]) versus non-NSU (medical [MICU], general surgical [SICU], trauma [TICU], cardiac [CCU], or mixed). The primary outcome measure was in-hospital mortality.

### Statistical Analysis

Continuous data are presented as means (X) and standard deviations (SD) or medians (Md) and interquartile ranges (IQRs) as appropriate based on the distribution of the data; categorical data are reported as proportions and 95% confidence intervals (CI). To evaluate of differences at the univariate level, the t-test for differences in means in normally distributed data or the Mann-U-Whitney test for non-normally distributed data; we used the χ^2^ or Fisher exact tests for differences in proportions. For the multivariable analysis, generalized estimating equations (GEE) were used to account for potential correlation in mortality rates among patients sampled within hospital clusters.^14^ Interactions were tested for all those significant variables retained in the GEE model. All statistical analyses were conducted using SPSS for Macintosh, Version 28. Significance was set at P < 0.05. Our reporting of observational data conforms with Strengthening the Reporting of Observational Studies in Epidemiology STROBE guidelines.^15^

## Results

A total of 32,047 patients met the inclusion criteria (10,010 admitted to NSU and 22,037 to non-NSU). The demographic data for this patient population are listed in Table 1. The national APACHE-IV model predicted a higher ICU mortality (14% vs 13%, P < 0.0001), in-hospital mortality (21% vs 19%, p < 0.0001), and hospital-LOS (9.9 days vs 9.6, p < 0.0001) for patients admitted to an NSU versus non-NSU (Table 2). However, when the actual outcomes were compared via univariate analysis post-stratification based on ICU designation, the ICU mortality (10% vs 11%, p < 0.01) and ventilator-free days (22 vs 24, p < 0.001) were lower in patients admitted to a NSU compared to non-NSU. On univariable analysis, the in-hospital mortality trend was lower in patients admitted to a NSU (15% vs 16%, p = 0.06). Importantly, all three outcomes were lower than those predicted by the APACHE-IV model. Hospital LOS was longer than non-NSU patients (10 days vs 8 days, p < 0.0001). These results are summarized in Table 3. In the multivariable regression analysis (GEE model) accounting for within-hospital effects and adjusted for known variables of poor outcome after brain injury (age, gender, race, and diagnosis), and the severity of illness APACHE-III score, admission to NSU was associated with lower actual mortality (OR 0.8; 95% CI: 0.7-0.9, p < 0.24) (Table 4). As expected, compared to TBI patients, the highest mortality occurred in ICH followed by SAH patients. AIS patients did not have significantly different in-hospital mortality compared to TBI (Table 4). We did not identify interactions among significant variables retained in the model.

**Table 1.** Demographic Data of the Study Divided Based on NSU Versus non-NSU Admission. Primary Cohort Characteristics (X = Mean, SD = Standard Deviation, Md = Median, IQR = Interquartile Range).

	NSU N = 10,010	Non-NSU N = 22,037	P-val
Age, Md (IQR)^a^	64.3 (16)	65 (17)	<0.0001
Gender, Female, N (%)^b^	5007 (50%)	10,152 (46%)	<0.0001
Race, White, N (%)^b^	6434 (64%)	16,298 (74%)	<0.0001
APACHE-III, Md (IQR)^a^	46 (31)	43 (30)	<0.0001
APS, Med (IQR)^a^	32 (20)	29 (25)	<0.0001
Neuro Score, Md (IQR)^a^	12 (12)	13 (12)	<0.0001
TBI, N (%)^b^	1360 (14%)	6015 (27%)	<0.0001
AIS, N (%)^b^	3783 (38%)	8956 (41%)	<0.0001
ICH, N (%)^b^	3389 (34%)	5400 (25%)	<0.0001
SAH, N (%)^b^	1478 (15%)	1666 (8%)	<0.0001

MD, Median; IQR, interquartile range; APS, advanced physiologic score.

a Mann-U-Wittney.

b Chi-square.

**Table 2.** Expected Outcomes Based on the APACHE III Prediction Models for the Study Population (X = Mean, SD = Standard Deviation).

	NSU N = 10,010	Non-NSU N = 22,037	P-val
ICU LOS, X (SD)	5 (2)	4 (2)	<0.0001
Hospital LOS, X (SD)	**9.9** (**4)**	9.6 (4)	<0.0001
ICU mortality, X (SD)	**14%** (**18)**	13% (18)	<0.0001
Hospital mortality, X (SD)	**21%** (**20)**	19% (20)	<0.0001
Vent. Free Days, X (SD)	23 (1.4)	24 (1.3)	<0.0001

**Table 3.** Actual Outcomes of the Study Population, Stratified as NSU Versus non-NSU (X = Mean, SD = Standard Deviation).

	NSU N = 10,010	Non-NSU N = 22,037	P-val
Hospital LOS, days X (SD)	10 (12)	8 (10)	<0.0001
ICU mortality, X (SD)	10%	**11%**	0.01
Hospital mortality, X (SD)	15%	**16%**	0.06
Vent. Free Days, X (SD)	**22 (7)**	24 (5)	<0.0001

**Table 4.** Results of Multivariate Regression Analysis Aimed at Assessing in-Hospital Mortality (OR = Odd Ratio, CI = Confidence Interval, Md = Median, IQR = Interquartile Range).

	OR	95% CI	P-Val
Age (>67 years)	1.1	0.99–1.2	0.2
Gender, male	0.9	0.8–0.9	.004
Race, white	0.9	0.8–1.1	0.5
APACHE-III	4.4	3.5–5.5	<.001
AIS*	1.1	0.9–1.3	0.2
ICH*	2.3	2.1–2.7	<.001
SAH*	1.4	1.2–1.7	<.001
**NSU** (ref Non-NSU)	**0**.**8**	**0.7–0.9**	.**02**

* Reference = TBI.

## Discussion

The purpose of this study was to compare the impact of NSU versus Non-NSU on conventional ICU-based benchmarks, specifically the in-hospital mortality. The main findings of our study were that critically ill brain-injured patients managed in dedicated NSU had: a) lower ICU mortality rates, b) lower proportion of poor outcome, and c) fewer ventilator-free days compared to patients admitted to non-NSU. Similarly, we observed a trend to lower hospital mortality approaching significance.

Our findings regarding ICU and hospital mortality were lower in patients managed in neurocritical care units than in general ICUs, however, our study results deviated from the predicted model, which estimated higher ICU and hospital mortality. One of the explanations for the difference in the predicted and actual outcomes could be because the APACHE III prediction model represents ICU day-1 mortality estimates, which shows the importance of reassessing the mortality risk daily in the ICU as they change depending on the acuity of the condition, the disease progression, and the available treatment options. Our outcomes were compared against the predicted outcomes of the APACHE III model, but several previous studies have criticized the model for underpredicting mortality, particularly in non-operative TBI patients.^10^

Diringer et al, in a retrospective study using Project Impact analyzed the data of 36,986 patients from 42 units (41 general ICUs and 1 Neuro-ICU) with a second NSU no participating in the database, found that not being in an NSU was associated with an increased in in-hospital mortality rate (OR 3.4; 95% CI, 1.65-7.6)^1^. Another retrospective study reviewed the clinical data and compared the outcomes between 1572 patients admitted to the ICU and 915 patients admitted to the NSU, in which authors found that the ICU mortality was significantly lower in the NSU compared to general ICU (4.7% vs 7.3%, p = 0.012).^13^ Likewise, Verelas and colleagues reported a mortality benefit for patients admitted to the neuroscience critical care unit. However, there was not statistical significance in patients with an acute head injury before and after the appointment of a fellowship-trained neuro-intensivist to direct the NSU.^16^

A retrospective study using a prospective database reviewed the data from 2381 patients admitted to the neuroscience critical care unit and found that the significant predictors of in-hospital mortality were APACHE Score and admission from another ICU type. Furthermore, the admission after the neurocritical care unit team was established was associated with significantly reduced in-hospital mortality (OR 0.7; 95% CI 0.5-1.0, p = 0.044). However, it was not associated with significant changes in long-term mortality.^17^ Our investigation found similar results, especially the decrease in in-hospital mortality for those patients admitted to a neurocritical care unit (OR 0.8; 95% CI: 0.7-0.8, p < 0.0001). We did not assess long-term mortality.

Two separate investigators reported a different outcome. Flaherty et al.,^18^ looked at long-term mortality following ICH in two large population-based cohorts assembled more than a decade apart. Mortality following ICH did not differ significantly between the two cohorts (1988 vs post-1998 through 2003), and it concluded that specialty ICUs did not impact mortality. Fogelholm and colleagues^19^ looked at long-term survival and predictors of death in patients with primary ICH. Hospital mortality and long-term survival markers were independent of specialty ICU, and Flaherty reached the same conclusion. Despite these findings, there has been an explosion of NSUs nationwide. The surge can be explained to some degree by the increase in highly specialized nurses and physicians in this field. More hospitals are shifting gears towards dedicated NSUs as the field of medicine becomes more specialized. The thought is that having dedicated staff will help decrease the mortality associated with critically ill patients.

A recent meta-analysis^2^ of mortality outcomes, including 26 non-randomized observational studies with 55,792 patients, showed that brain-injured patients receiving care in an NSU or by neurocritical care specialized staff had a 0.83-fold lower risk of mortality (17% relative risk reduction 95% CI, 0.70-0.97; P = 0.001) without any significant difference in LOS.

Notably, being managed in a neuro-critical care department does not seem to improve ventilation-free days in patients. Subjects treated in general ICUs spent more days free of mechanical ventilation, similar results as the predicted model. In contrast, Jeong and colleagues observed significantly fewer ventilator days in the NSU patients than general ICU patients (3.1 vs 4.2, P = 0.001).^13^

Moreover, we observed longer hospital length of stay, which aligns with the prediction model from APACHE III. Some of our thoughts regarding the reason behind this are: it can be due to the more management options and strategies available to those under the care of a specialized team that can predict any derangement and prolong the stay of the patients in the unit until they are further stabilized or the unavailability of step-down units for the patients in the NSU that prolong their discharge. Other retrospective studies had found similar results.^1^ Suarez et al.,^17^ found a decrease in both NSU (3.7 +/-3.4 vs 4.2+/-4; p < 0.001, CI, 0.2-0.8) and hospital LOS (8.4 +/-6.9 vs 9.9+/-8; p < 0.0001, 95% CI, 0.9-9.1) in those managed by a neuro-critical care team compared with those treated in a general ICU. Varelas et al.,^16^ also found positive results; NSU days went from 3.5 to 2.9 (p = 0.001, 95% CI, 0.2-0.9) before and after the appointment of a neuro-intensivist-led team model. We can attribute a couple of reasons to the decrease in NCCU and hospital LOS: the adhesion to guidelines, taking discharge criteria more rigorously, and mitigating complications.^7^

In terms of cost-effectiveness, Mirski et al^20^ showed lower costs and better outcomes in ICH patients after implementing of a Neuro-Critical Care Unit. In acute ischemic stroke patients, the more efficiency in resource utilization can be explained by the decreased hospital and NCCU LOS.^7^ The positive impact on the outcomes of having a dedicated ICU team is demonstrated in the literature**.**^1,2,7–9,13,16,17,21,22^ Moreover, some of these publications have also analyzed the intensity of staffing, finding much better outcomes with those units managed by high-intensity staffing as described usually as full-time intensivist.^1,17,20^

Our study has several limitations. First, it was a retrospective study of prospectively collected data, which could have limited the establishment of causal relationships. Because of the nature of the design, we could not control specific factors such as randomization and/or specific disease severity prediction scores. However, our multivariable regression models adjusted for highly predictive mortality scores in critically-ill populations (APACHE-III). Second, we could not stratify academic versus non-academic NSUs or NSUs based on volume, however we used robuts tatistical analysis to adjust for site-specific effects.^14^ Third, we could not control for the level and consistency of staffing (ie, 24-h physician in-house vs residents vs Advanced Practice Providers). Finally, we could not address other important outcome variables, such as functional state and quality of life, based on the inherent nature of the database used, which was not designed to capture those endpoints. This study also has important strengths. It has a large sample size collected across many institutions; while the study was retrospective, the data were prospectively collected for the APACHE IV database and data were extracted by professional data collectors for this study what gives strength to our data).

## Conclusion

Our findings demonstrated that brain-injured patients managed in neuro-critical care units by a dedicated team experienced lower ICU and hospital mortality rates with a reduced proportion of poor outcomes. However, these patients had prolonged ICU and hospital length of stay and required additional days of ventilator support. The presence of a dedicated neurocritical care team was associated with a mortality benefit, which we attribute to the multidisciplinary team's arrangement and well-trained personnel. This arrangement likely led to improved patient care through treatment standardization, enhanced monitoring processes, and reduced complications that may have gone undetected under the care of less specialized health workers.
